# Effects of metformin on survival outcomes of pancreatic cancer: a meta-analysis

**DOI:** 10.18632/oncotarget.18233

**Published:** 2017-05-26

**Authors:** Yi-Wei Dong, Yan-Qiang Shi, Li-Wen He, Xi-Yu Cui, Pei-Zhu Su

**Affiliations:** ^1^ The Second Clinical Medical School of Southern Medical University, Guangzhou 510282, Guangdong, China; ^2^ Department of Gastroenterology, The First People's Hospital of Foshan (Affiliated Foshan Hospital of Sun Yat-sen University), Foshan 528000, Guangdong, China

**Keywords:** pancreatic neoplasms, metformin, prognosis, meta-analysis

## Abstract

**Background and aim:**

Recent epidemiological studies indicated that metformin might improve the survival of various cancers. However, its benefit on pancreatic cancer was controversial.

**Methods:**

We performed this meta-analysis to investigate the benefit of metformin on pancreatic cancer. A comprehensive literature search was performed through PubMed, Cochrane Library and Embase. Relative risk (RR) and hazard ratio (HR) with 95% confidence interval (CI) were pooled.

**Results:**

The meta-analysis of 2 randomized controlled trials including181 pancreatic patients, revealed that metformin use was not associated with an improved overall survival at 6 months (RR=0.90, 95% CI=0.67-1.21), overall survival (HR=1.19, 95% CI=0.86-1.63) and progression-free survival (HR=1.39, 95% CI=0.97-1.99). But the meta-analysis of 8 cohorts, involving 2805 pancreatic patients with diabetes, demonstrated a favorable result with improved overall survival (HR=0.78, 95% CI=0.66-0.92).

**Conclusions:**

Observations in the cohort studies supported a favorable role of metformin while the data from randomized controlled trials did not support that. Therefore, more high-quality RCTs are warranted.

## INTRODUCTION

Pancreatic cancer is the fourth leading type of cancer death in both male and female in the United States. Despite the improved surgical technique and new chemotherapeutic regimens, the outcome of pancreatic cancer remains poor because of high aggression and treatment resistance. Currently, the overall 5 year survival rate is 7%. And more than half of cases are diagnosed at an advanced stage with unresectable metastatic disease, whose 5 year survival rate is only 4% [1].

Diabetes mellitus (DM), a worldwide metabolic disorder, is identified as one of strong epidemiological risk factors for pancreatic cancer, besides smoking, age and chronic pancreatitis etc [2, 3]. The majority of pancreatic cancer patients were diagnosed with either new-onset type 2 diabetes or impaired glucose tolerance [4, 5]. The mechanism of the association between pancreatic cancer and DM has not been clearly elucidated yet. Evidence shows that hyperinsulinemia, insulin resistance, hyperglycemia, chronic inflammation and elevated circulating insulin-like growth factors causing by DM may promote the cancer growth [6, 7]. Furthermore, recent epidemic studies and reviews have demonstrated that pancreatic cancer patients with DM might be associated with worse survival than patients without DM [8–10], but it is too premature to draw a definitive conclusion. Metformin, a relatively inexpensive and well tolerated oral anti-diabetestic drug for the treatment of T2DM, has raised worldwide attention for its potential anti-tumorigenic effects. The molecule mechanisms of metformin's anti-cancer activities mostly rely on its ability to inhibit the LKB1-AMPK-mTOR signaling pathway and the cell division, to promote apoptosis and autophagy and down-regulate the circulating insulin [11–15]. Therefore, it is reasonable to regard metformin as a potentially effective and safe agent for various cancers. Accumulating evidence has shown that metformin use may be associated with a good prognosis in cancer with diabetes [16–18].

Preliminary studies and reviews have demonstrated that metformin use is correlated with reduced pancreatic cancer risks and improved pancreatic cancer outcomes [19–24]. However, recent randomized controlled trials (RCTs) and one large-scale cohort study showed no existence of such correlation [25–27]. Notably, analysis methods of some studies were criticized due to time-related bias [28]. Here, we systematically performed a meta-analysis to explore the metformin exposure on the survival in patients with pancreatic cancer.

## RESULTS

### Study selection and characteristics

**Figure 1:**
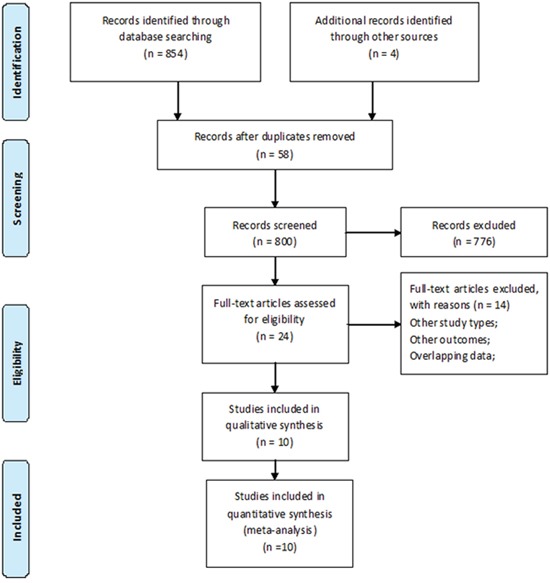
The flow diagram for the included studies

A total of 858 records were retrieved through databases and other sources (Figure 1). According to the predefined inclusion and exclusion criteria, 2 RCTs, including 144 patients without diabetes and 37 patients with diabetes, and 8 cohorts, including 2805 patients with diabetes were finally included in our meta-analysis [19, 23–27, 29–32]. The main characteristics of the identified studies were summarized in Tables 1 and 2.

**Table 1:** Characteristics of included cohort studies

Study, year, country	Definition of exposure	Stage of cancer	Metformin	Nonmetformin	Median age (years)	Survival analysis	Adjusting variables	Duration and follow-up (year, months)	NOS scores
Sadeghi *et al*, 2012, USA.	Ever/never, regardless of the dose and duration of metformin use and other combinational therapies they had received.	Resectable(67)/unresectable(124)/metastatic(111)pancreaticadenocarcinoma	117 with pre-cancer diagnoses of DM	185 withpre-cancer diagnoses of DM	64.0±8.7	OS	Disease stage, serumCA-19-9 level, tumor size(cm), tumor site(tail), performance status	2000-2009, 11.4.	6
Hwang *et al*, 2013, United Kingdom.	1. Metformin use around the time of PAC diagnosis (between 6 months prior and 1 month after); 2. Without prior (i.e., 6 months before PAC diagnosis) exposure to metformin	Advanced pancreatic adenocarcinoma	247 with pre-cancer diagnoses of DM	269 with pre-cancer diagnoses of DM	72.5±10	OS	Age, sex, duration of diabetes, presence of diabetic complications, history of pancreatitis, Charlson index, BMI, GFR, smoking at the time of diagnosis, history of insulin use, history of sulfonylurea use, history of thiazolidinedione use, and HbA1c	2003–2010, NR.	6
Kim *et al*, 2014, Korea.	NR	Resectable/unresectable/metastatic(220)	111 with DM	317 with DM	NR	OS	ECOG performance status, CA 19-9, cancer stage, body massindex (BMI) and number of organ involvement	2005-2010, NR.	4
Lee *et al*, 2015, Korea.	Cumulative duration of metformin use at more than 1 month after diagnosis.	Resectable/locally advanced/metastatic	117 with pre-cancer diagnoses of DM	120 with pre-cancer diagnoses of DM	66 (34–85)	OS	ECOG performance status, tumor size, tail involvement, CA 19-9 level, and cancer stage.	2005-2013, 10.3	5
Ambe *et al*, 2016, USA.	Ongoing use and never used	Resectable pancreatic adenocarcinoma cancer	19 with a preoperative diagnosis of DM	25 with a preoperative diagnosis of DM	68 (40–88)	OS	NR	1986-2013, 19	7
Kozak *et al*, 2015, USA.	Continued use before and after surgical resection	Resectable pancreatic adenocarcinoma cancer	13 with a preoperative diagnosis of DM	102 with a preoperative diagnosis of DM	69 (37–86)	OS, DFS	N stage, age, margin status, adjuvant radiation, and gemcitabine	1998-2013, 11.23	4
Choi *et al*, 2016, Korea.	NR	Advanced pancreatic cancer	56 with DM	297 (127 with DM)	59.6	OS	All patients: performance status, cancer extent and weight loss during first-line therapy; DM subsets: None.	2003-2010, 10.2	6
Chaiteerakij *et al*, 2015, United Kingdom	Different definitions of exposure were analyzed.	Resectable(284)/Locally advanced(354)/metastatic(341)Pancreatic cancer	366 with DM	614 with DM	67.4	OS	Age, sex, disease stage, body mass index, and diagnosis year group	2000-2011, 9.26	7

Abbreviations: DM: diabetes mellitus; NR: not reported; OS: overall survival; DFS: disease-free survival; NOS: Newcastle-Ottawa Scale.

**Table 2:** Characteristics of included randomized controlled trials

Study	Study design	Intervention	Stage of cancer	Metformin	Placebo	Median age (years)	Survival analysis	Adjusting variables	Duration and follow-up (year, months)	Jada scores
Reni *et al*, 2015, Italy.	Open-label, randomized, phase II trial	PEXG every 4 weeks in combination or not with 2 g oral metformin daily	Metastatic pancreatic cancer	31	29	Metformin:64 (42–75); Placebo:63 (44–73).	PFS at 6 months, PFS, OS	Age, CA 19-9, Karnofsky performance status, lymph nodes metastasis, peritoneum metastasis, SNP rs11212617, adiponectin, IL-6.	2010-2014, NR.	5
Kordes *et al*, 2015, Netherlands.	A double-blind, randomized, placebo-controlled phase 2 trial	Received either oral metformin or placebo twice daily.	Advanced pancreatic cancer	60	61	Metformin:64 (45–78);Placebo:65 (44–79).	OS at 6 months, OS, PFS	Tumor stage and diabetic state	2010-2014, 28.1m.	7

Abbreviations: NR: not reported; OS: overall survival; PFS: progression-free survival.

The involving studies were published between 2012 and 2016 with the sample size ranging from 60 to 980 patients. The country where the studies were conducted included Korea (n=3), USA (n=3), United Kingdom (n=2), Italy (n=1) and Netherlands (n=1). HRs were calculated by results of adjusted multivariate analysis except the HR of OS in one cohort and the HR of PFS in another cohort were only available in univariate analysis [24, 32]. The definitions of metformin exposure were reported in 6 cohort studies and the definitions varied across studies. With regard to the quality assessment, the NOS scores of individual cohort studies ranged from 4 to 7 with median of 5.63 and the Jadad scores of two RCTs were 5 and 7 respectively.

### Metformin and survival of pancreatic cancer patients in RCTs

**Figure 2:**
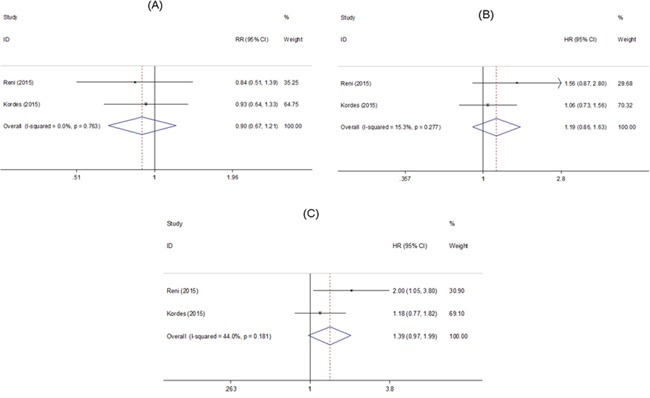
Forest plot of the effect of metformin on pancreatic cancer in randomized controlled trails HR: hazard ratio; RR: relative risk; CI: confidence interval. **(A)** Association between metformin effect and overall survival at 6 months; **(B)** Association between metformin effect and overall survival; **(C)** Association between metformin effect and progression-free survival.

A total of 2 RCTs comprising 181 patients reported HRs for OS at 6 months and PFS [25, 26]. Metformin use was not associated with an improved OS at 6 months (RR=0.90, 95% CI=0.67-1.21, Figure 2A) with low heterogeneity (*P_h_* =0.763, I^2^=0.0%, Figure 2A), OS (HR=1.19, 95% CI=0.86-1.63, Figure 2B) with low heterogeneity (*P_h_*=0.277, I^2^=15.3%, Figure 2B) and PFS (HR=1.39, 95% CI=0.97-1.99, Figure 2C) with moderate heterogeneity (*P_h_*=0.181, I^2^=44.0%, Figure 2C).

### Metformin and survival of pancreatic cancer patients in cohort studies

**Figure 3:**
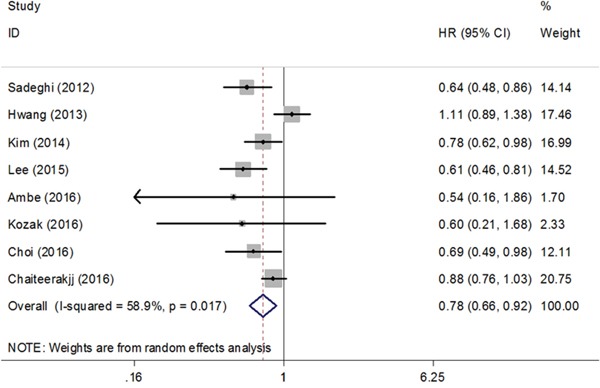
Forest plot of the effect of metformin on overall survival of pancreatic cancer in cohort studies HR: hazard ratio; CI: confidence interval.

A total of 8 cohort studies comprising 2805 patients reported HR for OS [19, 23, 24, 27, 29–32]. Metformin use was shown to be beneficial to the increased OS (HR=0.78, 95% CI=0.66-0.92, Figure 3) with moderate heterogeneity (*P_h_*=0.017, I^2^=58.9%, Figure 3). In the subgroup analysis, the HR of Asian countries was 0.70 (95% CI=0.60-0.83) and the HR of Western countries was 0.84 (95% CI=0.67-1.07) (Supplementary Figure 1).

### Sensitivity analysis and publication bias of cohort studies

The leave-one-out analysis revealed that metformin use remained significant with the omission of each individual cohort study in turn. Through calculating the pooled HRs via sensitivity analysis, we found that the study by Hwang et al. contributed to heterogeneity in the meta-analysis of cohort studies [19]. The pooled HRs after exclusion of this study was 0.77 (95% CI=0.69-0.85, *P_h_*=0.236, I^2^=25.2%). The pooled HRs (95% CI) after adjusting for stage (n=5), performance status (n=3), age (n=4), BMI (n=3), CA19-9 (n=3), resectable cancer (n=3) and advanced cancer (n=3) were 0.78 (0.66–0.92), 0.69 (0.59–0.80), 0.90 (0.81–1.01), 0.91 (0.81–1.01), 0.69 (0.59–0.80), 0.75 (0.56–1.00) and 0.84 (0.62-1.15) (Table 3). No obvious publication bias was detected by Begg's funnel plot shape or Egger's test (p=0.218) (Supplementary Figure 2).

**Table 3:** Pooled hazard ratios of overall survival in diabetic pancreatic cancer with and without metformin

Type of estimate	Number of studies	Metformin	Nonmetformin	Effects model	Pooled HR (95% CI)	I^2^, %	P value
All studies	8	1046	1759	Random	0.78(0.66-0.92)	58.9	0.017
Estimates adjusted for stage	5	724	1338	Fixed	0.78(0.70-0.86)	45.4	0.120
Estimates adjusted for performance status	3	345	622	Fixed	0.69(0.59-0.80)	4.1	0.352
Estimates adjusted for age	4	737	1302	Fixed	0.90(0.81-1.01)	47.2	0.128
Estimates adjusted for BMI	3	724	1200	Fixed	0.91(0.81-1.01)	60.6	0.079
Estimates adjusted for CA 19-9	3	345	622	Fixed	0.69(0.59-0.80)	4.1	0.352
All estimates for resectable cancer	3	129	314	Fixed	0.75(0.56-1.00)	0.0	0.773
All estimates for advanced cancer	3	435	618	Random	0.84(0.62-1.15)	74.8	0.019

Abbreviations: CI: confidence interval; HR: hazard ratio; BMI: body mass index.

## DISCUSSION

This meta-analysis, including 2 RCTs and 8 retrospective cohort studies, comprehensively investigated the effect of metformin on survival outcomes of pancreatic cancer patients. The results of RCTs, which included 144 patients without diabetes and 37 patients with diabetes, were insignificant in OS at 6 months, OS and PFS. Meta-analysis of 8 cohorts including 2805 patients with DM demonstrated significant results that metformin exposure increased the OS. With regard to the pooled HRs for OS, no obvious publication bias existed. Furthermore, the benefit of metformin on OS was still robust across sensitivity analyses except for those including studies adjusted for age, BMI and all estimates for resectable cancer or advanced cancer.

The results of meta-analysis of retrospective cohorts supported the beneficial effects of metformin exposure on pancreatic cancer patients with diabetes. Metformin inhibited DNA synthesis and proliferation of pancreatic cancer cells close to the desmoplastic reaction by down-regulating receptor-PI3K-mTOR signaling pathway [33–36]. By down-regulating vascular endothelial growth factor b signaling pathway, metformin enhances anti-tumor effects of resveratrol on pancreatic cancers [37]. Additionally, metformin could inhibit the activation of insulin-like growth factor and insulin-like growth factor receptor through the reduction of insulin, and reduce the concentration of insulin receptor substrat 1 to inhibit the PI3K-Akt signaling pathway, which would influence the action of mTOR [38, 39]. The mitochondrial complex I is a promising target in cancer cells and the decreased ATP production caused by the inhibition of complex I probably contributes to the anti-tumor effects [40, 41]. Furthermore, metformin would have a therapeutic effect on the metabolic abnormalities caused by diabetes which might have an adverse influence on response to cancer treatments [42].

However, the latest large cohort study and meta-analysis of two RCTs demonstrated different outcomes [25–27]. In the study reported by Reni et al, despite metformin caused endocrine and metabolic modification, the modification was not correlated with favorable PFS at 6 months, PFS, and OS of pancreatic cancer patients. Besides, the insulin concentration, insulin receptor expression on tumor tissue and genetic susceptibility to metformin metabolism had no significant correlations with clinical outcomes [25]. In the RCT conducted by Kordes et al, metformin use did not have a statistically significant association with prolongation of survival in pancreatic cancer patients with diabetes, however, metformin use was statistically significantly correlated with survival in patients with locally advanced pancreatic cancer, when stratified by clinical stage [26]. Modest anti-tumor effect and small study sizes may account for ineffectiveness of metformin in advanced pancreatic cancer patients [43]. Besides, RCTs included pancreatic cancer patients with or without diabetes while all the cohorts involved patients with diabetes. RCTs excluded patients with history of using metformin before. As is known, diabetes is a poor prognostic factor for cancer and metformin can control the progression of diabetes. The large cohort of 980 patients with diabetes by Chaiteerakij et al did not find the benefit of metformin use by different definitions of exposure either [27]. Additionally, they illustrated that the different definitions of exposure would have unintended influences on the estimated results, which corresponds to the concerns about time-related biases in the observational studies by Suissa et al [28]. Of interest, they found a statistically significant difference in survival among patients with local advanced cancer. These patients received metformin before cancer diagnosis. This conclusion is similar to the finding of the RCT by Kordes et al [26]. These observations suggest that future trials should focus on the protective effects in patients with locally advanced cancer. The time-biases would come from the time-fixed analysis. Some retrospective studies performed analysis only using a conventional Cox proportional hazards regression model, and most of them suggested beneficial effects of metformin. However, with this analysis, patients were commonly categorized as ever/never use classification and HRs were computed at a specific time, such as the time of diagnosis. The patients who did not receive metformin at the timing but some time after were automatically classified as ever use group. This kind of patients lived long enough to have the chance to receive metformin after previous cancer treatment and their survival time might result from better baseline health other than metformin. Therefore, the ignorance of the timing of initiation probably causes time-related bias and overestimation of metformin effect. To minimize this bias, the time-varying covariate Cox model treated metformin use as time-dependent variables and the patient is not classified in the ever use group until metformin use is initiated.

All in all, several reasons account for the negative result. First, some observational studies were methodologically inaccurate by using a time-fixed analysis and would lead to overestimation of metformin effects for pancreatic cancer patients. Second, the metformin might be beneficial to pancreatic cancer patients with diabetes rather than pancreatic cancer patients without diabetes, and the hypothetical anti-neoplastic activity might be probably based on the concurrent diabetes. Third, the heterogeneity in cancer stage might contribute to the discrepancies in findings between RCTs and cohort studies. Among 8 cohort studies, six studies included patients in resectable cancer stage. However, the two RCTs were conducted in late stage patients, such as metastatic and advanced cancer. Forth, as for the molecule mechanism, although *in vitro* experiments showed anti-neoplastic activities of metformin on cancer cells, the concentration in patient's neoplastic tissue fail to achieve a level as stable and sufficient as it in cells to cause energetic action [44]. As an anti-cancer agent, maybe metformin with a higher dose than the anti-diabetic dose is required. Additionally, metformin would have potential antagonism effect against chemotherapy [45]. Metformin acting cell autonomously can either increase or decrease reactive oxygen species generation through which most of chemotherapeutic drugs work.

Several limitations in this meta-analysis need to be considered. First, the administration of metformin exposure, including dosage, duration and timing of metformin initiation, were varied across included studies. However, relevant sensitivity analysis was not conducted due to the limited data. Second, the different characteristics of pancreatic cancer, including pathological types, tumor size and clinical stages, would contribute to high heterogeneity. Third, therapeutic schedules for pancreatic cancer and their effects on survival were not described clearly, which would affect estimations of metformin on cancer survival. Fourth, only one study adjusted the use of other anti-diabetic drugs including sulfonylureas and insulin, which might result in underestimation of benefits of metformin [19]. Fifth, we were supposed to extract multivariate HR to minimize the effects of confounders. However, we selected the univariate HR rather than multivariate HR in some special occasions. The univariate HR of OS was selected from the study by Choi et al, because the multivariate HR was for all patients while the univariate HR was for diabetes patients only and the latter was what we needed according to our aim [24]. Additionally, the univariate HR of PFS was chosen in study by Kozak et al because of the lack of precise multivariate HR [32]. Although sensitivity analyses were conducted in accordance with adjusted variables, the number of studies included in each sensitivity analysis was small and probably contributed to overestimation and underestimation of the treatment effects. Sixth, one abstract was included in our meta-analysis in order to obtain adequate survival information, while the details of the baseline and outcome were unavailable [29]. Seventh, our meta-analyses were based on summarized data rather than data from individuals, which might result in overestimation of treatment effects.

With regard to the association between metformin and the outcome of pancreatic cancer patients with diabetes, whether patients were exposed to metformin before cancer diagnosis requires attention. The RCT of Kordes et al showed patients who achieved a decrease in insulin concentrations in the metformin group had improved survival than those who did not achieve decreased insulin [26]. However, insulin reductions in the placebo group were not associated with a survival benefit, indicating that whether the insulin level are associated with metformin effects warrants further studies. In addition, more pharmacodynamic assessments of metformin effects on mitochondrial glycolytic metabolic function should be explored in future researches, such as insulin receptors and the organic cation transporters.

In conclusion, our meta-analyses showed that observations in the cohort studies supported a favorable anti-cancer role of metformin while data from RCTs did not support this role. Additionally, metformin exposure might benefit pancreatic cancer patients with concurrent diabetes. To determine the effect of metformin on survival after pancreatic cancer diagnosis, more high-quality RCTs are warranted.

## MATERIALS AND METHODS

### Literature research

This meta-analysis follows PRISMA guidelines (Supplementary File 3) [46]. PubMed, Cochrane Library and Embase were systematically searched up with search strategies based on following terms used in PubMed: (“Metformin”[Mesh] OR (metformin OR biguanides OR “hypoglycemic agents”)) AND (“Pancreatic Neoplasms”[Mesh] OR ((pancreatic OR pancreas) AND (neoplasms OR cancer OR carcinoma))) AND (“Prognosis/mortality”[Mesh] OR (prognosis OR prognostic OR outcome OR survival OR mortality)). Any restriction including language, human research or study design was not permitted. Besides, manual searching of references in identified studies and relevant reviews was conducted to retrieve every potential article.

### Inclusion/exclusion criteria

Two authors independently reviewed the candidate studies, and discrepancies were resolved by discussion. We selected eligible studies according to the predefined inclusion and exclusion criteria. Inclusion criteria were: (1) RCTs or cohort studies with controlled group; (2) studies investigating the association between metformin use and the prognosis of pancreatic cancer patients; (3) analysis of survival outcome including overall survival (OS) or progress-free survival (PFS); (4) sufficient information to estimate relative risk (RR) and hazard ratio (HR) with 95% confidence interval (95% CI). Exclusion criteria were: (1) study types including case report, review, case series, editorial and letter; (2) studies without sufficient data to estimate RR or HR with related 95% CI; (3) language other than Chinese and English; (4) nonhuman researches.

### Data extraction and quality assessment

Data of identified studies was extracted by two investigators independently. The latest study with more abundant data was included in meta-analysis when the data overlapped cross studies. The following information was extracted: author, year of publication, country of study, definition of exposure or intervention, sample size, age, survival analysis, adjusting variables, duration, follow-up and data of survival analyses. If several estimations were conducted in one study, the most powerful result was selected (i.e., the multivariate regression will be given priority, and the univariate regression was superior to the unadjusted Kaplan-Meier analysis). If several definitions of exposure or population were reported in one study, data from the definition which was more similar to the remaining studies was extracted and other data was collected for sensitivity analyses. The quality assessments were applied using the Newcastle-Ottawa Quality Assessment Scale (NOS) for cohort studies and Jadad scale for RCTs by two reviewers independently [47, 48]. Discrepancies were discussed and resolved in the process.

### Statistical analysis

RRs and HRs along with their 95% CIs were gained directly from included studies or from estimation based on methods by Parmer et al, resulting in a conservative estimate of the significance level [49]. The significance of the pooled HR and RR were determined by Z-test, and the level of statistical significance was established as *P*<0.05 [50]. The heterogeneity among studies was checked by the Q test and Higgins I-squared statistic [50, 51]. The Mantel–Haenszel method based on fixed effects model would be performed to calculate the pooled HRs on the condition that *P* value for the heterogeneity test was greater than 0.05 [52]. Otherwise, the random effects model based on DerSimonian and Laird method would be used [53]. The sensitivity analysis was conducted by omitting individual studies. Furthermore, considering the variations in the covariates, we conducted sensitivity analyses by calculating pooled HRs with estimates adjusted for certain confounders, including stage, performance status, age, body mass index (BMI) and CA 19-9. Sensitivity analyses were also conducted in all estimates for resectable cancer and advanced cancer. Publication bias was evaluated by Begg's funnel plot and Egger's test (*P*<0.05 was considered a significant publication bias) [54, 55]. The meta-analyses were performed using Stata version 12.0 software (Stata, College Station, TX, USA).

## SUPPLEMENTARY MATERIALS FIGURES AND TABLE




