# Supplementary material for: Effects of metformin on survival outcomes of pancreatic cancer: a meta-analysis
Source: Oncotarget. 2017 May 26;8(33):55478–88. doi: 10.18632/oncotarget.18233 (PMC5589674; doi:10.18632/oncotarget.18233)
Supplement: Supplementary file 1 [file oncotarget-08-55478-s001.pdf]

Effects of metformin on survival outcomes of pancreatic cancer: a meta-analysis

SUPPLEMENTARY INFORMATION

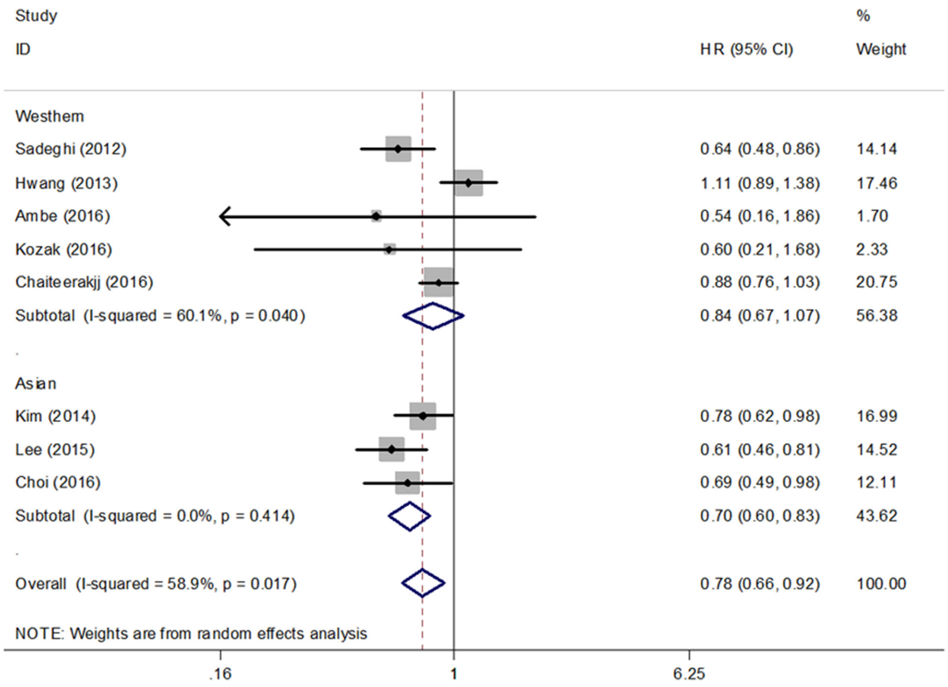

Supplementary Figure 1: Sub-group analysis of the effect of metformin on overall survival of pancreatic cancer in cohort studies. HR: hazard ratio; CI: confidence interval.

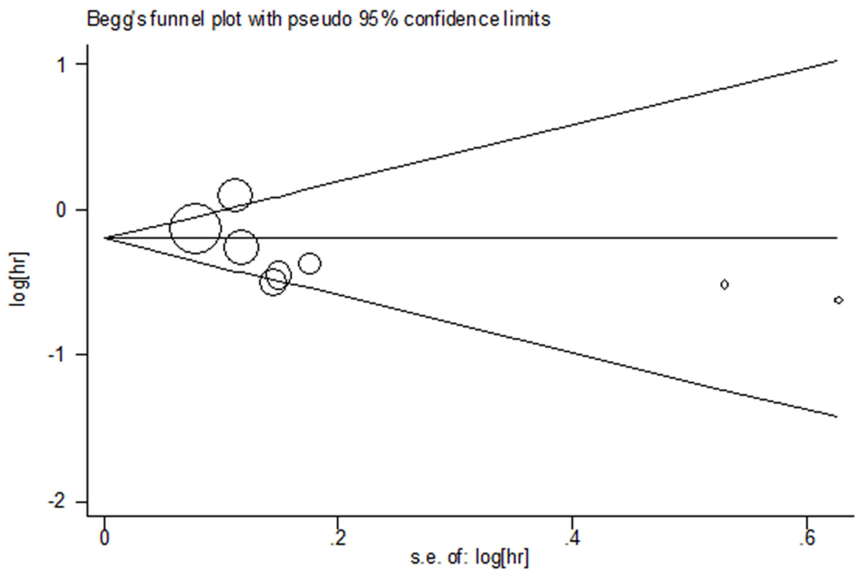

Supplementary Figure 2: Begg's funnel plots for publication bias test for cohort studies.

Supplementary File 3: PRISMA Checklist

See Supplementary File 1
